# “Chill packs” self-administered olanzapine as psychiatric harm reduction for methamphetamine-induced psychosis: a narrative review of the existing evidence base

**DOI:** 10.1186/s12954-026-01475-1

**Published:** 2026-06-04

**Authors:** Isabelle Ruedisueli, Carla Marienfeld, Casey Tiefenthaler, Allison Lee, Alicia Harvey-Vera, Steffanie Strathdee, Joseph R. Friedman

**Affiliations:** 1 School of Medicine, University of California, San Diego, USA; 2 Department of Psychiatry, University of California, 200 W Arbor Dr., San Diego, CA 92103 USA; 3 Department of Emergency Medicine, University of California, San Diego, USA; 4 Department of Internal Medicine, University of California, San Diego, USA; 5 Department of Pharmacy, University of California, San Diego, USA

**Keywords:** Methamphetamine use disorder, Antipsychotics, Psychiatric harm reduction, Self-administration, Psychosis

## Abstract

The prevalence of methamphetamine use disorder has risen across the United States and many other countries, which has led to a concomitant rise in methamphetamine-induced psychosis (MIP). A promising and novel approach to address MIP can be found in ‘chill packs’, a psychiatric harm reduction intervention for people using methamphetamine. Chill packs consist of several low-dose olanzapine tablets (an antipsychotic medication) that can be self-administered in the event of methamphetamine-induced insomnia, anxiety, paranoia, delusions, or other dysphoric symptoms. One recent study assessed the effectiveness of an implementation of chill packs by the San Francisco Department of Health and found that repeat psychiatric emergency visits dropped by 32% after two-months and 13% after six-months from receipt of a chill pack. While these findings are promising, more research is needed to evaluate the efficacy and generalizability of this intervention. Here we provide a narrative review which synthesizes indirect evidence and contextual information relevant to this intervention, including: the epidemiology of MIP, clinical data supporting the use of olanzapine versus other antipsychotics for MIP, evidence for as-needed outpatient antipsychotic use, as well as qualitative and other data describing the potential for misuse of olanzapine and other antipsychotics. Overall, the literature supports that the prevalence of MIP is rising sharply, causing increased psychiatric burden. This is also linked to overdose risk among patients that use illicit fentanyl or other sedatives to “come down” after periods of heavy methamphetamine use. Olanzapine has demonstrated comparable efficacy for treating MIP compared to other antipsychotics with enhanced short-term sedative effects, despite a higher burden of long-term metabolic side effects. Olanzapine has very little documented misuse risk, although other agents in its class, especially quetiapine, have higher street value. Qualitative research highlights that some groups already self-administer olanzapine as a ‘trip killer’ to manage distressing psychiatric side effects from illicit drug use. Overall, we find substantial contextual evidence supporting further trials to evaluate chill packs as a harm-reduction intervention for MIP and provide recommendations for research needed to fill gaps in the evidence for this promising emerging intervention.

## Introduction

Mortality and morbidity from methamphetamine use is a growing public health crisis in the United States and other nations globally [1, 2]. Although opioid-related deaths have been decreasing in the United States, this is not true for methamphetamine-involved deaths, which continue to rise—a marker of the rising usage and importance of this class of drugs [1, 3]. Furthermore, the majority of the disease burden associated with stimulant use arises from chronic health effects, such as accelerated cardiovascular disease or psychiatric morbidity, which are also increasing rapidly. 

**Fig. 1:**
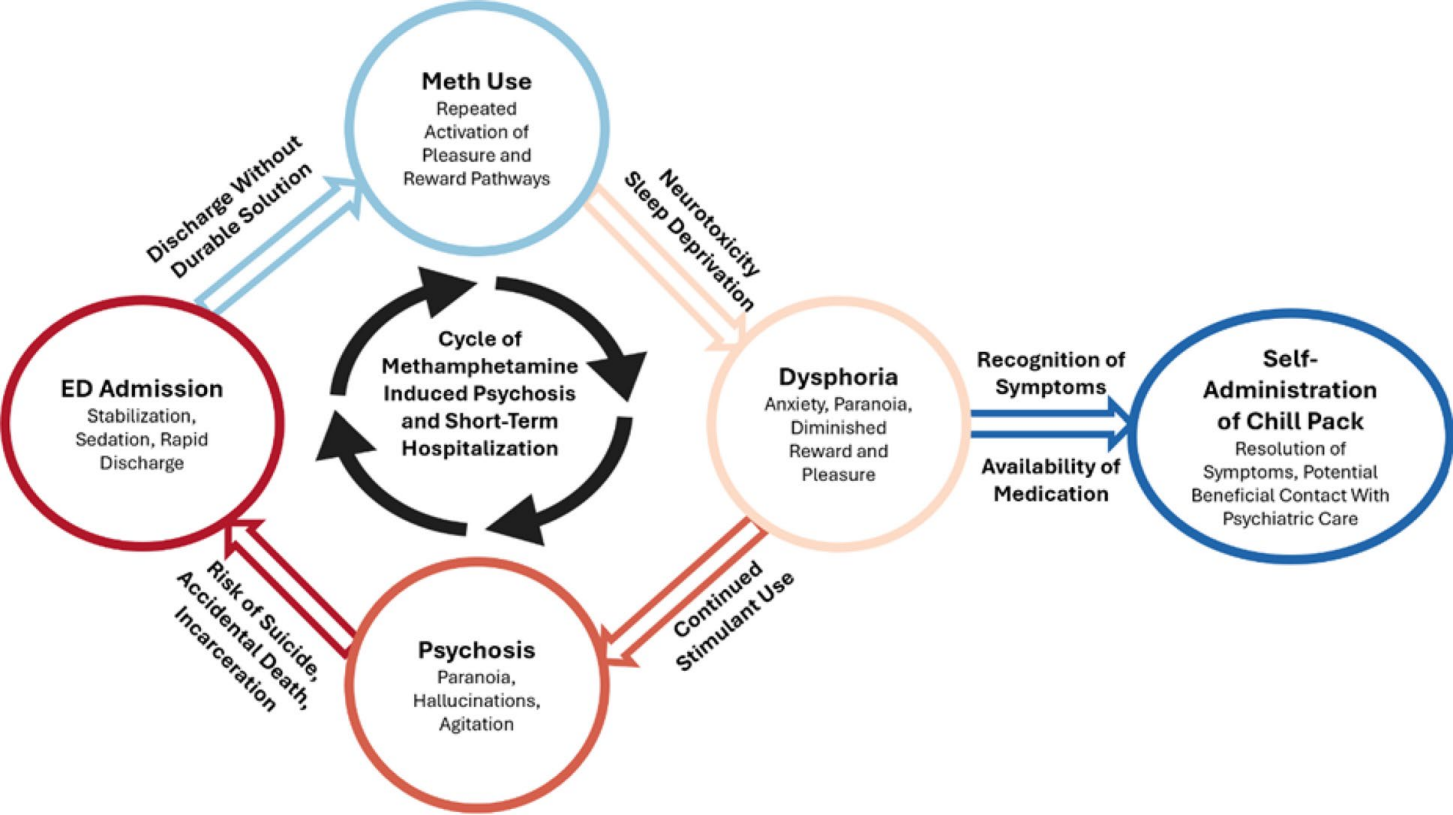
A diagram depicting the cycle of methamphetamine induced psychosis and resulting short-term hospitalizations, as well as the proposed role for the chill pack intervention

It is well-documented that prolonged methamphetamine use precipitates psychotic symptoms and other psychiatric sequalae for a high proportion of people that regularly use the drug [4]. Between 2015 and 2019 the rate of methamphetamine-involved psychiatric hospitalizations increased by 68% nationally, and the Western United States was the region of the country most affected, though rates in other regions are now also rising as the substance spreads in popularity nationally [2]. Methamphetamine-induced psychosis (MIP) represents a particularly high-risk phase in the natural history of methamphetamine use disorder, associated with increased mortality and legal consequences, and it represents a key opportunity for intervention (Fig. 1). Methamphetamine use is initially highly euphoric, providing activation of neural circuits responsible for sensations of reward and pleasure, leading many individuals to use it in a repeated fashion over a several-day period [4–7]. Nevertheless, with each subsequent administration the euphoric effects diminish, while prolonged neurotoxic effects and sleep deprivation, lead to a predominantly dysphoric state, with anxiety and paranoia [5, 6].

Qualitative interviews with individuals that use methamphetamine suggest that at the dysphoric stage of the usage cycle, many individuals experience a strong desire to use sedating substances to achieve sleep [6, 8]. On some occasions, this serves as a motivation for the use of illicit fentanyl—placing people that primarily use stimulants at high risk of opioid overdose—or the use of diverted quetiapine, benzodiazepines, or other sedatives [5, 8–10]. Other individuals continue to use methamphetamine, seeking a return to euphoric and rewarding effects, and develop worsening paranoia, anxiety, and in some cases psychotic symptoms including, delusions and auditory, tactile, and/or visual hallucinations. Individuals experiencing MIP are vulnerable to death by suicide, homicide, or other injuries, and are also at high risk of legal consequences [6, 11, 12]. In many cases MIP results in short-term hospital stays, via law enforcement, EMS activation, or the voluntary seeking of care. During these hospital admissions, individuals experiencing MIP are typically given short courses of antipsychotic and other sedating medications [13, 14]. After metabolizing methamphetamine, and sleeping, many individuals experience rapid resolution of psychotic symptoms and are discharged. These admissions are often frequent, cyclical, and result in a considerable time and financial burden for the individuals involved and the healthcare system and community [13].

A promising and novel approach can be found in ‘chill packs’ [15], a psychiatric harm reduction intervention for people who use methamphetamine. The concept entails providing individuals with methamphetamine use disorder—and no underlying primary psychotic condition—with a limited number of olanzapine tablets that they can self-administer in the event of methamphetamine-induced insomnia, anxiety, paranoia, delusions, or other dysphoric symptoms. The intervention relies on the premise that individuals using methamphetamine can detect the onset of dysphoria or early psychosis and self-administer a medication to achieve sleep and ‘break the cycle’ of MIP, preventing short-term hospitalization (Fig. 1).

Although this approach is highly promising, the direct evidence base supporting it remains limited; a single study from the San Francisco Department of Health demonstrated real-world effectiveness [15]. In this study, published in July 2024, Coffin et al. showed that the provision of chill packs was associated with a 32% reduction in repeat psychiatric emergency visits after two-months, and by 13% after six-months. However, at 12-months, no difference was found. These findings suggest short-term effectiveness as a harm-reduction intervention which attenuates over time due to either depletion or loss of medication [15]. Nevertheless, should this approach prove broadly effective, chill packs could be scaled up as a harm reduction intervention through frequent contacts with community-based providers and outreach-based distribution. This intervention therefore has the potential to reduce the burden of psychiatric services needed for methamphetamine-induced psychosis and may improve outcomes among individuals with methamphetamine use disorder.

Although this early evidence is encouraging, more research is needed to evaluate the effectiveness of this intervention. Here, we provide a narrative review of the literature describing contextual information and indirect evidence supporting the approach, including related contextual information, such as: the epidemiology of MIP, the comparative efficacy of olanzapine vs. other antipsychotics for this condition, evidence supporting as-needed (often referred to as *PRN*) use of antipsychotics in the outpatient setting, as well as qualitative and other data describing the misuse potential of these medications.

## Search strategy and selection criteria

This narrative review synthesizes the existing literature relevant to the “chill packs” intervention and the treatment of MIP. We searched PubMed and Google Scholar for articles published through October 2025. Search terms included combinations of “methamphetamine,” “psychosis,” “olanzapine,” “antipsychotics,” “harm reduction,” and “self-administration.” We prioritized randomized controlled trials comparing antipsychotic efficacy for MIP, epidemiological data on methamphetamine-related hospitalizations, and qualitative research regarding the self-management of substance use sequelae. Articles were restricted to the English language. Because of the nascent nature of the chill packs intervention in the field, and the breadth of related contextual information spanning various epidemiological and clinical topics, a narrative approach was chosen to synthesize key contextual and indirect evidence surrounding this novel intervention, rather than serving as an exhaustive systematic review of all available literature [16].

To ensure comprehensive coverage of this multifaceted topic despite the non-systematic design, an iterative, purposive sampling strategy was employed. Initial literature gathering focused on identifying the most salient, highly cited foundational studies within each defined sub-domain [17]. These primary papers served as methodological anchors for bidirectional snowball citation tracking. We systematically reviewed the reference lists of these core articles (backward snowballing) and examined subsequent literature citing them (forward snowballing) to identify additional relevant works. This iterative search and extraction process was conducted independently for each distinct thematic category and was sustained until conceptual saturation was achieved—defined operationally as the point at which further literature searches yielded redundant thematic insights and no novel empirical or theoretical frameworks emerged [18].

## The epidemiology of methamphetamine use and related psychosis

Stimulant use, particularly methamphetamine, and its associated psychiatric sequelae represent an escalating public health crisis affecting many world regions [19]. As recent global monitoring highlights, the illicit market for synthetic stimulants has expanded to unprecedented levels, characterized by high availability, diversified manufacturing, and increased potency [19]. While consumption trends vary between countries, the morbidity and mortality associated with regular stimulant use—including substance-induced psychosis—have placed a strain on healthcare systems in many countries [13]. Prolonged and intense use frequently precipitates distressing psychiatric symptoms, including paranoia, auditory and visual hallucinations, and severe agitation [13, 14]. As international markets for synthetic stimulants expand, addressing the acute psychiatric consequences of use has become a paramount priority for harm reduction and clinical responses in many settings.

In the United States, the availability, purity, and overall use of methamphetamine have surged in recent years, driving a sharp increase in associated health consequences. Survey data from 2024 estimated that 2.3 million people aged 12 and older had used methamphetamine in the past year [20]. This rise in use has corresponded with a dramatic escalation in mortality; when excluding deaths co-involving fentanyl, the rate of primary methamphetamine-involved overdose deaths experienced a nearly 7-fold increase from 2010 to 2024 [21]. Although historically concentrated in the West [1], methamphetamine use has increasingly diversified across all major regions and populations of the United States, disproportionately impacting American Indian and Alaska Native individuals, as well as unhoused populations [1, 22].

This intensification of use has also catalyzed a large increase in the rate of acute psychiatric emergencies, specifically methamphetamine-induced psychosis (MIP). Between 2015 and 2019, US psychiatric hospitalizations involving methamphetamine use rose by 68% [2]. By 2024, there were over 560,000 methamphetamine-related emergency department (ED) visits nationwide, which more frequently required chemical restraints and resulted in longer lengths of stay compared to non-substance-related visits [23]. Rising ED utilization rates for the treatment of MIP have also been implicated in hospital overcrowding and provider strain among already overburdened emergency and acute care settings, underscoring the urgent need for outpatient and harm-reduction interventions [24, 25].

Beyond the US, the high prevalence of stimulant use extends to multiple global regions. North America and East and South-East Asia continue to be the largest and most active methamphetamine markets, accounting for the largest share of global methamphetamine seizures in 2023 [19]. In East and South-East Asia, methamphetamine remains the drug of major health concern, accounting for the majority of people in substance use disorder treatment [19]. In Scandinavia, amphetamine is the most commonly injected drug, and amphetamine consumers are overrepresented at syringe services programs [26–28]. Other regions are also experiencing significant impacts: Oceania has the second-highest prevalence of amphetamines use behind North America [5, 19, 29], and Mexico and Canada have also seen escalating health harms from amphetamines [8, 30, 31]. Furthermore, emerging markets have seen rapid increases in methamphetamine seizures, particularly in pockets of Europe, Africa, the Near and Middle East, and South-West Asia [19]. Given these regional patterns, harm reduction interventions for stimulant users, such as chill packs may have global significance. Existing harm reduction infrastructure, such as needle exchange programs, could serve as strategic and effective distribution sites for providing self-administered olanzapine, and are already working with vulnerable individuals in many areas where injected or smoked amphetamine use is prevalent.

## Choice of antipsychotic: olanzapine vs. other agents for MIP

Most literature assessing the efficacy of antipsychotic medications for MIP has primarily relied on randomized control trials, largely conducted in inpatient settings over four-to-six week periods. These studies typically measure reductions in positive and negative symptoms of psychosis using scales such as the Positive and Negative Syndrome Scale (PANSS) ([32–38]. Patients generally receive medications once or twice daily and are monitored routinely for symptom improvement, time to recovery, dropout rates, and adverse effects including extrapyramidal symptoms [32–38]. These trials mainly examine antipsychotic medications prescribed for regular, scheduled use rather than the use on the as-needed basis employed in the chill packs intervention. Nevertheless, they still provide valuable evidence into which medications may be more effective, achieve faster symptom reduction, and have fewer or less severe adverse effects, informing the treatment of MIP with as-needed medications.

Across studies, olanzapine has consistently demonstrated efficacy in the treatment of MIP, and has been among the most tolerable antipsychotics with fewer side effects (despite more severe metabolic side effects from long-term use) [39]. Compared to other antipsychotics, olanzapine has a broader receptor interaction profile [40, 41]. Like most other second generation antipsychotic mediations olanzapine exerts antipsychotic effects via blockade of dopamine D2 receptors and Serotonin 5-HT2A receptors [40]. It is also among the most sedating antipsychotic medications, related to its effects on histaminergic and alpha receptors.

Olanzapine was compared directly to haloperidol in one of the earliest head-to-head comparisons for treating MIP by Leelahanaj et al. (2005), which demonstrated that although both medications effectively resolved psychosis, patients taking olanzapine experienced less frequent and less severe extrapyramidal symptoms, as measured by the Simpson-Angus scale (SAS) and Barnes Akathisia Scale (BAS) [32]. A 2018 study found that olanzapine and haloperidol were equally effective in treating stimulant-induced mental disorders, as assessed by the Brief Psychiatric Rating Scale (BPRS) which includes subscales for anxiety and depression, lack of vitality, cognitive disturbance activation, and hostile suspicion [33]. Subsequent studies similarly found comparable overall efficacy between olanzapine and haloperidol, with olanzapine demonstrating faster symptom improvement (mean 6.3 days relative to 9.4 days for haloperidol), fewer adverse effects, and earlier resolution of psychotic symptoms on the Clinical Global Impressions–Severity scale [33]. Notably, olanzapine proved superior at alleviating symptoms of anxiety and depression, lack of vitality, and cognitive disturbances during the four-week study period, despite showing no difference in total BPRS scores at the study’s completion [33].

Several other antipsychotic medications have also been evaluated for the treatment of MIP. For example, haloperidol was compared to quetiapine, an atypical antipsychotic with a similar but distinct receptor-binding profile to olanzapine [34, 42, 43]. Both quetiapine and haloperidol were effective at treating psychosis, showing no significant differences in cure rates (defined as PANSS scores < 33 post-treatment) or the time to cure [34]. Adverse effects across the mesocortical and mesolimbic, extrapyramidal, anticholinergic, adrenergic, and antihistamine-related categories were comparable between the two medications [34]. Risperidone and aripiprazole, both atypical antipsychotics, which vary in their affinity for specific dopaminergic and serotonergic receptor subtypes targeted, were also compared in MIP [44]. In a 2012 study, both agents reduced positive and negative symptoms as measured by the Scale for Assessment of Positive Symptoms (SAPS) and Scale for Assessment of Negative Symptoms (SANS), with risperidone demonstrating a greater reduction in hallucinations, thought disorder, anhedonia, and inattentiveness than aripiprazole [35]. Wang et al. (2012) reported similar findings showing that both medications equally decreased psychotic symptoms [38]. Notably, symptoms of akathisia and agitation were significantly greater in patients taking aripiprazole relative to risperidone [38].

Srisurapanont et al. (2021) performed a meta-analysis including some of the aforementioned randomized control trials. Several of the studies suggest that olanzapine has a faster onset and more rapid resolution of symptoms, with fewer and less severe extrapyramidal symptoms, however comparisons with other antipsychotic medications beyond haloperidol remain limited. This review found that olanzapine and quetiapine may have the best efficacy for treating MIP; however the overall quality of the evidence was low due to variability across trials, persistent risk of bias, and inconsistencies in positive symptom data [36]. This review emphasized the paucity of literature guiding the treatment of MIP and called for further investigation on the topic. In absence of high-quality clinical trial data establishing a clear consensus, we concur with Srisurapanont et al. (2021) that olanzapine or quetiapine represent reasonable first-line agents for the short-term treatment of MIP. Olanzapine offers additional advantages, including potent antipsychotic effects at relatively low doses, clinically useful sedation in agitated states, minimal potential for misuse, and the option for transition to a one-month long-acting injectable formulation. Therefore, olanzapine constitutes a reasonable first-line choice for harm-reduction–oriented interventions such as chill packs, while we acknowledge that multiple antipsychotics may be appropriate depending on patient-specific factors.

## Benefits and side effects of olanzapine for short-term, low dose use to treat MIP

Olanzapine use is associated with several well-characterized potential side-effects including weight gain, metabolic changes, and less commonly, extrapyramidal symptoms including akathisia, tardive dyskinesia, or neuroleptic malignant syndrome [39, 45–48]. Additional side effects of olanzapine include sedation and hypotension [45, 46, 49]. There is no clear evidence of dangerous interactions between olanzapine and stimulants such as methamphetamine, although there are potential concerns regarding this class of medication and QT prolongation [40]. Some of the effects of olanzapine, such as hypotension and sedation, which can be considered side effects in some contexts, are often therapeutic in the context of stimulant toxidromes that can cause hypertension and agitation [33, 40].

Following oral administration, peak plasma concentrations are typically achieved within approximately 6 h, with the elimination half-life between 21 and 45 h; steady-state plasma concentrations are generally reached after one week of daily dosing [50]. Olanzapine metabolism is not significantly affected by alcohol consumption, but literature suggests that tobacco smokers likely need a higher dose than nonsmokers, as hydrocarbons in cigarette smoke deactivate the medication via Cytochrome P450 1A2 induction [51]. While olanzapine has a low potential for toxicity when prescribed as monotherapy, case reports have documented cardiorespiratory depression with high-dose intravenous or intramuscular olanzapine when administered together with potent central nervous system depressants such as parenteral benzodiazepines [52]. Olanzapine is usually prescribed at doses ranging between 5 mg-40 mg, and is administered once or twice daily for patients with schizophrenia or bipolar disorder [41]. In acute settings, oral or intramuscular administrations are commonly used to decrease agitation with common side effects including sedation, dizziness, and orthostatic hypotension [46, 53–55]. Lower doses (< 5 mg), have also been investigated in other patient populations for the short-term management of behavioral disturbances in patients with dementia and for appetite stimulation and weight gain for patients with cancer and anorexia nervosa [56–60].

In the first study of the chill packs intervention, psychiatric practitioners prescribed oral olanzapine in the form of 5 mg tablets, with instructions for participants to self-administer the medication on an as-needed basis when experiencing dysphoric symptoms associated with methamphetamine use [15]. Characterizing the side-effect profile of this specific dosing strategy—specifically, low-dose, oral and intermittent use—would ideally require clinical trials conducted under comparable conditions. However, data describing adverse effect rates in this context remain limited. However, the side effects for short-course use of low-dose, oral olanzapine can likely be extrapolated from existing analyses of acute low doses in healthy individuals, low-dose oral use in the emergency setting, and low-dose intramuscular administration in patients with schizophrenia.

Among healthy individuals, the acute administration of olanzapine—typically at a single oral dose of 10 mg [61–63]—has been utilized to characterize its baseline effects on sleep architecture, metabolic regulation, and reward system modulation. For instance, one study of 13 healthy participants demonstrated that while a 10 mg dose did not alter the macroscopic structure or number of sleep cycles, it significantly increased electroencephalography (EEG) theta power across both sexes [61]. This neurophysiological modulation is clinically relevant: theta activity during rapid eye movement (REM) sleep facilitates emotional memory consolidation, while robust slow-wave sleep is often compromised in psychotic spectrum disorders such as schizophrenia [64]. Consequently, these findings in healthy controls provide a mechanistic rationale for how olanzapine might successfully ameliorate the severe sleep disruptions frequently observed in MIP [61, 65].

In another study of eight healthy individuals, a single 10 mg oral dose of olanzapine was administered alongside a high-fat meal to assess its impact on postprandial triglycerides [62]. Hourly blood sampling over an eight-hour period revealed no significant changes in free fatty acid or triglyceride levels, prompting the authors to conclude that D2 receptor antagonism is unlikely to alter postprandial lipids. The metabolic profile of olanzapine was further characterized in a separate trial where a 10 mg oral dose was followed by a delayed glucose bolus at 4.25 h [63]. Compared to placebo, olanzapine administration resulted in a sustained increase in plasma glucose levels and reduced glucose effectiveness, as measured by a frequently sampled intravenous glucose tolerance test. While insulin sensitivity remained acutely unaffected, olanzapine was associated with decreased cortisol, lower fasting free fatty acids, and elevated prolactin concentrations [63].

In a separate double-blind, crossover study, eight healthy subjects received a single 5 mg oral dose of olanzapine, and their reward system modulation was assessed through functional magnetic resonance imaging during a monetary incentive task [66]. Compared to the placebo condition, reward-related brain activation was significantly reduced in the ventral striatum, anterior cingulate, and inferior frontal cortex. The authors concluded that olanzapine dampens the assignment of incentive salience within these dopaminergic regions. This mechanistic finding provides a neurobiological rationale for why olanzapine may have utility in treating substance misuse: by dampening incentive sensitization, it may counteract the reward system dysregulation that is a well-documented consequence of heavy methamphetamine use.

In a multi-center study conducted across 16 psychiatric hospitals in Spain, researchers evaluated the acute administration of oral olanzapine in 278 patients admitted for agitation and psychosis, over two-thirds of whom had a primary diagnosis of schizophrenia [55]. Within this cohort, 148 patients received olanzapine monotherapy, while 15 were treated with olanzapine combined with other antipsychotics. For those receiving oral olanzapine, the mean initial dose was 11.54 ± 4.87 mg (range: 2.5–20 mg). Approximately one-third of the patients required a repeated pharmacological intervention, such as a supplementary dose of olanzapine, haloperidol, or levomepromazine. During the period from hospital admission to discharge (whether discharged home or transferred to inpatient care), patients exhibited significant reductions in agitation across validated measures, including the CGI-S, ACES, and PANSS-CE scales. Furthermore, the safety profile was favorable: only 3.4% of patients treated with olanzapine reported adverse effects—including bradycardia, dry mouth, sedation, hypertension, and hypotension—with no extrapyramidal symptoms observed [55]. Although the median observation period of 1.83 h was significantly shorter than the six hours required for the drug to reach peak plasma concentrations, these findings nevertheless suggest a low incidence of immediate adverse effects following acute olanzapine administration.

Literature demonstrates that, when feasible, oral atypical antipsychotics are at least as effective as intramuscular (IM) formulations in managing acute psychotic agitation, supporting their use as first-line therapy [67]. Nevertheless, clinical data derived from IM administration provides valuable insights into the safety and efficacy of the acute, low-dose olanzapine proposed for the chill packs intervention. In a multi-center study evaluating IM olanzapine for acute agitation in 270 patients with schizophrenia across 14 sites in Europe and Africa, patients receiving 5, 7.5, or 10 mg doses demonstrated significantly greater clinical improvement at two hours compared to those receiving 2.5 mg, based on median PANSS-EC scores [54]. Over a 24-hour observation period, patients in the placebo group were significantly more likely to require additional rescue injections. Furthermore, all olanzapine dose groups exhibited greater sustained improvement than the placebo group across multiple validated scales (PANSS-EC, ABS, ACES, and BPRS Total)—with the singular exception of the 2.5 mg group on the BPRS Positive scale [54].

Crucially, the safety profile for these low-dose cohorts was generally highly favorable. Over the 24-hour treatment period, hypotension occurred in only two of 48 patients receiving 2.5 mg, and two of 45 patients receiving 5 mg. Similarly, emergent akathisia was observed in only two of 42 participants in the 5 mg group, and was entirely absent in the 2.5 mg cohort. Overall, these findings underscore a markedly low incidence of adverse effects associated with the acute administration of low-dose olanzapine.

Taken together, the existing literature indicates that short-term, low-dose olanzapine exhibits a favorable safety profile, minimizing the severe metabolic risks typically associated with chronic administration, or the extrapyramidal side effects more common with other antipsychotic medications. Furthermore, its acute physiological and neurobiological effects—namely sedation, mild hypotension, and the dampening of reward-system incentive salience—may be uniquely well suited to counteract the agitation and dopaminergic dysregulation characteristic of methamphetamine toxicity. When combined with its potential to restore disrupted sleep architecture, these converging lines of evidence provide a compelling potential robust rationale for the choice of olanzapine for the chill packs intervention. Ultimately, extrapolating from both healthy volunteer and acute psychiatric data, intermittent, low-dose oral olanzapine emerges as a safe, tolerable, and mechanistically targeted strategy for managing the immediate sequelae of methamphetamine-induced psychosis.

## Evidence describing the misuse or diversion risk of olanzapine

Although antipsychotics are not traditionally considered as drugs of misuse, some limited emerging evidence supports the notion that their non-prescribed use and diversion have increased. Reports from case studies, case series, ambulance data, emergency departments, and poison information centers suggest that individuals who misuse antipsychotics are predominantly male, with a history of substance use disorders and frequently incarcerated in prison, psychiatric, or addiction treatment settings [68]. Misuse occurs via several routes including oral, intravenous, or inhalational administration [68–70].

Atypical antipsychotics are not considered to have a high-risk of misuse due to their dopamine antagonism which contradicts the dopamine reward pathways implicated in addiction [71]. Therefore, understanding the intent for their misuse is of interest. Malekshahi et al. (2015) interviewed 25 individuals who were receiving addiction treatment: 67% reported misusing antipsychotics to recover, 25% took the medication to enhance the effects of other substances, and 20% used antipsychotics for experimental purposes [69]. Additionally, when patients reported misusing atypical antipsychotics, the 96% reported using the atypical antipsychotic, quetiapine (96%) compared to olanzapine (28%) [69]. The higher percentage of quetiapine misuse aligns with its many street names including “quell”, “Susie-Q”, “baby heroin”, and “Q-ball” (when taken with cocaine or heroin), and participants have described combining this medication with opioids, cocaine, alcohol, and opioids [69, 72, 73]. Several pharmacological factors may explain quetiapine’s higher misuse profile. Its greater sedative and anxiolytic properties are attributed to effects at alpha-adrenergic and histamine receptors, while its lower potency at dopamine receptors relative to other atypicals may contribute to reinforcing effects [70, 71, 74]. These qualities may contribute to its misuse and help explain a report comparing the adverse drug events of different second-generation antipsychotics. In a 2019 review of the FDA Adverse Event Reporting System, Evoy et al. found that quetiapine had a disproportionate number of misuse-related events compared to olanzapine, aripiprazole, and risperidone [75]. Along with its pharmacological abilities, quetiapine’s misuse may be due to its supply. The drug has been more commonly available in US carceral settings compared to other medications in the same class. Overall, quetiapine is prescribed more often than olanzapine in the US, and low-dose quetiapine has become increasingly prescribed “off-label” for anxiety or sleep symptoms in patients without a severe mental illness or psychiatric disorder [76, 77]. The rise in off-label prescriptions may be increasing accessibility and availability of the medication in illicit markets.

The misuse of olanzapine has been largely characterized through case reports and appear to mostly involve medication obtained directly from doctors and used differently than prescribed, rather than reflecting a robust illicit market for the drug [78]. For instance, in one case report, a 53-year-old with psychotic depression was prescribed 20 mg/day for psychotic symptoms. The patient elected to double this dose, without consulting her physician, to alleviate her depression and reported feeling generalized anxiety if she took less than this amount [79]. In a separate case report, a 48-year-old was prescribed 50 mg/day of olanzapine with citalopram for three years. When the olanzapine was discontinued due to metabolic concerns in the context of a diabetes diagnosis, the patient experienced anxiety, insomnia, and dysphoria that she felt would only improve with resumption of the medication [80]. Finally, a 25-year-old with bipolar disorder and substance use disorder reported taking 40 mg of olanzapine to get “a buzz” and relax. He used the medication to augment or alter his substance use, blunt “jitters” from cocaine use, “come down” from stimulant use, and experience euphoria when combining the medication with alcohol [81]. The ‘misuse’ of olanzapine, as embodied through these case reports, includes patients increasing or sustaining high dosages to combat feelings of anxiety. Further, like quetiapine, the final case study highlights that olanzapine may be used in combination with substances, including the facilitating the stimulant de-escalation period [82]. This notion of olanzapine as an effective “comedown” agent is also endorsed by a specific subgroup who describe olanzapine as the ultimate “trip terminator” in qualitative data [82].

Olanzapine’s efficacy for counteracting toxicity from substance use is potentially supported by qualitative data collected among individuals who experiment with novel psychoactive substances (NPS) [82]. Pro-drug blogs and online platforms allow users to share their experiences, offer support, and give advice to others trying novel drugs like synthetic cannabinoids, psychedelic phenethylamines, synthetic cathinones, and tryptamines [82, 83]. From November 2012–2013, eight pro-drug websites were analyzed by Global Public Health Intelligence Network software to understand how users self-managed psychotomimetic effects [82]. Of the antipsychotics discussed in these forums, olanzapine was identified as the most commonly used medication to end “bad trips,” followed by quetiapine, risperidone, aripiprazole, haloperidol, and clozapine [82]. Blog posts lauded olanzapine’s ability to “terminate”/“modulate” the acute unwanted effects of their substances as well as various withdrawal symptoms including anxiety, depression, dysphoria, and insomnia [82]. Designated the “ideal” drug for this purpose, most individuals would take 5–10 mg/day of olanzapine for several days after an illicit substance “trip”, to treat undesired effects, with a few taking up to 50 mg/day [82]. In one post, a user spoke to the comfort of having the medication available, not only for himself but for others: “olanzapine is excellent. The pharmacological profile says it all, and I have terminated a friend’s horrific and violent trip with this pharm. [….] Also I might add, Zyprexa [the brand name for olanzapine] has been a real lifesaver before when I was having a horrible trip from psilocybin. I will never trip again without having some [olanzapine] (or Seroquel) on-hand…” [82]. Another user emphasized their confidence in olanzapine to quell unwanted symptoms from methamphetamine use: “…My favourite pick of all is olanzapine, a very strong, new style, atypical. It is very very sedating, [.]this is good, as you trying to kill a trip […]. For dealing with amphetamine or methamphetamine olanzapine wins hands down in my book” [82]. This qualitative data, albeit limited, provides a window into a subculture of individuals already utilizing olanzapine in a self-administered manner that they experience as preventing psychiatric harms from their substance use [83].

## Parallels to the implementation of community narcan programs

Other harm reduction models involving administration of medications in community settings may offer insights that are potentially useful for the implementation of chill packs. Community distribution of naloxone has been widely embraced by communities, distributed by organizations, and adopted by individuals who use drugs [84–87]. Naloxone is an opiate antagonist that was first used to reverse opioid overdose in clinical settings but now is distributed widely through injectable and nasal spray forms [84, 88, 89]. Beginning in 2010, access to naloxone increased steadily through various initiatives, and by July 2017 every state had passed laws increasing naloxone access [86]. While state legislation varied, laws largely ensured the dispensing of naloxone without individual prescriptions, increased use by emergency medical services, increased distribution and education by healthcare providers, and civil and criminal immunity for individuals who administered naloxone to others [86, 89]. With these changes in effect, states with naloxone access laws that ensured a wider distribution had a 14% lower incidence of opioid-overdose mortality compared to other states without naloxone access laws [89]. Alongside the increase in legislation was a considerable push to place naloxone in the hands of individuals who use opioids and their family and friends, as opposed to limiting access to healthcare professionals [88]. This movement built on the success of community-based opioid overdose prevention programs [84, 90]. In an early Massachusetts study which followed individuals who had completed overdose education and nasal naloxone distribution (OEND) programs from 2002 to 2009, of the rescue attempts later reported, 87% were performed by persons who were actively using, in treatment, or recovering from use, while only 13% were among ‘nonusers’ [84]. Introduction of these programs also was associated with a 27–46% reduction in unintentional opioid related overdose death rates in the nineteen Massachusetts communities in which they were implemented [84].

Naloxone’s positive trajectory and increased adoption can also be seen through interviews of individuals who use opioids. In New York City, thirty-nine people who use opioids attended an overdose training and received injectable or nasal naloxone [85]. They were interviewed later about their experience administering naloxone in an overdose and 38 of 39 individuals reported successfully reversing an overdose. Neale et al. argues that this result demonstrates that people who use opioids can not only be educated to respond to overdoses, but can respond with a high level of practical and social competency [85]. In a similar protocol, 18 individuals (with 17 of18 reporting current use of opioids, heroin, or methamphetamine) received training and opioid overdose rescue kits from a community organization and were later interviewed about their views on naloxone and experience using naloxone on a peer [86]. Half of participants reported administering naloxone on more than one occasion and felt naloxone was readily accessible and effective at reducing overdoses. They were highly satisfied with the training program and felt prepared to administer naloxone in an overdose [86]. Naloxone training programs have also facilitated the transfer of information between peers. Between 2004 and 2005, twenty-five individuals who inject drugs participated in a prevention and distribution program, and one-third later shared the training information with their peers [87]. Some individuals even referred others to the program or required knowledge on how to administer naloxone as part of their “house rules” [87].

The overall high acceptability and effectiveness of community-based naloxone distribution programs, implemented mainly via people who use drugs, suggests that chill packs could have similar high acceptability and uptake among communities of individuals that use stimulants.

## Conclusions and recommendations for future research

 A summary of the indirect and contextual evidence supporting further study of the chill packs intervention is shown in Table 1. The epidemiological data describing MIP highlights that the condition is rising in prevalence. MIP-related psychiatric hospitalizations are also increasing across the country, and treating MIP through avenues other than costly ED and acute care settings is increasingly necessary. Furthermore, interventions that can prevent dysphoric symptoms from progressing to frank psychosis in the context of methamphetamine use can provide meaningful benefits for the quality of life of individuals that use these substances. The clinical data supporting the use of olanzapine vs. other antipsychotics for this condition, is imperfect, yet olanzapine is certainly among the best choices for this purpose, if not the single best short-term medication. Olanzapine has been studied in the treatment of MIP and described as having a quicker onset and more rapid resolution of psychosis while causing less extrapyramidal symptoms than other antipsychotics, but more trials are warranted to make more definitive conclusions. In comparison to quetiapine, olanzapine also appears to have limited street value and minimal misuse risk. Nevertheless, even the ‘misuse’ of olanzapine noted in the literature is largely in-line with the goals of the chill packs intervention; olanzapine has been endorsed as an efficacious “trip terminator” for individuals who use psychoactive substances, allowing safer hallucinogenic and stimulant use with reduced risk of psychosis. Low-dose olanzapine has been shown to effectively reduce psychotic symptoms and offer helpful sedative effects. Building on the experience of other harm reduction interventions such as naloxone, with increasing uptake in community settings, we argue that self-administered olanzapine could also become readily adopted and embraced by individuals who use drugs and desire to use them more safely. Overall, we find considerable indirect contextual evidence supporting further trials to evaluate the chill packs intervention.

**Table 1:** Evidence summary from domains of indirect and contextual evidence supporting further study of the chill packs intervention

Evidence domain	Study types	Key findings	Relevance to chill packs
Clinical efficacy of antipsychotics in MIP	RCTs (inpatient, scheduled dosing) and case reports	Olanzapine, quetiapine, haloperidol, and risperidone reduce psychotic symptoms in MIP; olanzapine has faster onset and fewer EPS vs. haloperidol	Supports clinical feasibility that olanzapine can alleviate acute MIP symptoms
Comparative tolerability	Head-to-head trials, meta-analysis, RCT, and observational studies	Olanzapine associated with fewer and less severe EPS, including at low doses in the emergency setting; aripiprazole is associated with higher rates of akathisia and activating properties	Favorable short-term tolerability supports use in unsupervised, intermittent setting
Effects of single dose olanzapine in healthy individuals	Experimental, single dose studies	One-time administration of olanzapine (10 mg) in healthy individuals not associated with significant disruption in sleep architecture, lipid metabolism, or overall reactivity	One-time, low doses of olanzapine may not disrupt sleep, metabolism, or reactivity, bolstering its intermittent use for MIP
Misuse of olanzapine vs. other antipsychotics	Cross-sectional, observational analysis and case reports	Quetiapine reported as misused more than olanzapine in interviews; olanzapine used in self-directed fashion by individuals wishing to de-escalate symptoms from illicit drug use (especially psychedelics or amphetamines) or self-increasing doses to alleviate anxiety	Olanzapine misused less than quetiapine and misused for alleviation of symptoms from substances may signal limited diversion risk
Qualitative reports describing self-guided use of olanzapine	Retrospective, observational qualitative analysis	Olanzapine reported as most used antipsychotic to manage psychomimetic effects from experimentation with psychoactive substances	Designation as the “ideal trip terminator” by online reports may support olanzapine’s well-suited nature for treatment of MIP
Other self/community administered harm reduction interventions (Naloxone)	Time-series analysis and qualitative and observational analyses	Majority of rescue attempts for opioid overdoses undertaken by individuals who use drugs; individuals who use drugs embrace naloxone for opioid overdose and pass along information to peers	Like naloxone, olanzapine may mitigate the acute, harmful effects of substance use despite the medications differing in their effects on mortality. Communnity-based distribution may be feasible.

Despite the encouraging initial data and indirect evidence, direct empirical support is currently limited to a single observational analysis [15]. This study demonstrated the real-world effectiveness of chill packs, reporting a 32% reduction in repeat psychiatric emergency visits at two months. However, this finding reflects effectiveness in only one specific urban setting. To validate this intervention as a standard of care, large-scale randomized controlled trials are required.

Future research must also broaden beyond efficacy to address implementation and generalizability. Implementation science studies are needed to determine optimal distribution models across diverse geographic settings where MIP is a rising challenge—ranging from rural communities to varied urban centers—and to navigate the logistical challenges of “as-needed” prescribing in outreach contexts. The Coffin et al. study was embedded in a relatively high-resource psychiatric emergency department setting, which may require considerable modification for adaptation elsewhere. Additionally, given that the protective effects in the pilot study waned by 12 months, research should target strategies to sustain engagement and medication retention over time. An outreach approach maximizing frequent patient contacts with repeat chill pack provision may be particularly promising, albeit resource intensive. Community-based naloxone provision experienced particularly high uptake once the burden of individualized prescription was lifted. At the moment, olanzapine provision is restricted to instances of personalized prescription, however this may be an area for future study and innovation.

Additional study is also needed to assess usage patterns among patients with consistent access to chill packs for MIP. Although chill packs have so far only been studied as a short-term intervention, if distribution is scaled up, patients may access them repeatedly, or may seek long-term olanzapine prescriptions after noting benefits for MIP after taking a chill pack. If patients fall into a pattern of daily, extended use, the risk of metabolic side effects may become a primary concern, and providers should consider transitioning them to a different, more metabolically favorable antipsychotic medication. Nevertheless, it is likely that in practice some patients will end up on olanzapine for long-term use in the context of chronic stimulant induced psychosis—just as among patients with schizophrenia and other primary psychotic conditions—and in those cases clinical judgement is needed to manage risks and benefits to patients. Further study of optimal chill pack treatment duration and frequency is needed to optimize outcomes.

Finally, quantitative inquiries must be paired with rigorous qualitative research to center the voices of people who use drugs. Understanding user perspectives on packaging, dosage, and perceived barriers to self-administration is essential for refining the intervention. Much like the expansion of community-based naloxone, which empowered peers to reverse opioid overdoses, chill packs may have the potential to empower individuals to manage their own acute stimulant intoxication safety. However rigorous evaluation is still needed to demonstrate the efficacy, generalizability, and implementation guidelines for this promising intervention.

## Data Availability

No datasets were generated or analysed during the current study.
